# The discovery of Bevacizumab. An historical reappraisal

**DOI:** 10.1007/s10238-025-01857-y

**Published:** 2025-10-17

**Authors:** Domenico Ribatti

**Affiliations:** Department of Translational Biomedicine and Neuroscience, University of Bari, Bari, Italy

**Keywords:** Angiogenesis, Anti-angiogenesis, Bevacizumab, Tumor progression, Tumor resistance, VEGF

## Abstract

In 1997, the monoclonal antibody A4.6.1 was humanized to create Bevacizumab (Avastin, Genentech), an antibody suitable for clinical trials. In February 2004, Bevacizumab was approved in a randomized double-blind phase III study in which was administered in combination with bolus IFL (irinotecan, 5FU, leucovorin) chemotherapy as first-line therapy for previous untreated metastatic colorectal cancer. In 2020, the EMA approved the first biosimilar of Bevacizumab for the treatment of multiple types of cancer. The administration of Bevacizumab became popular among ophthalmologists for different ocular diseases. However, in most cases of cancers, including breast, melanoma, pancreatic and prostate cancer, Bevacizumab failed to increase survival. Despite impressive performances in animal models, however, inhibitors are not performing nearly as well in humans. The limitations of applying Bevacizumab are attributed to drug resistance, metastasis promotion and reduced delivery of chemotherapeutic agents, resulting from the dramatic decrease in tumor vasculature.

## Background of experimental evidence

In 1971, Judah Folkman predicted the existence of a tumor angiogenesis factor (TAF) that would induce the growth of blood vessels in tumor and developed the concept of “anti-angiogenesis” as a potential treatment of cancer due to “the prevention of new vessel sprouts from penetrating into early tumor implant” [1]. Beginning in the 1980’s, industry exploited the field of anti-angiogenesis for creating new therapeutic molecules in angiogenesis-dependent diseases.

Vascular endothelial growth factor (VEGF) pathway is the most important signaling pathway in the angiogenesis process, and many inhibitors have been developed to block the action of VEGF and its receptors (VEGFRs), with more than ten approved drugs for various tumors being used in clinical practice.

Napoleone Ferrara and his colleagues at Genentech were the first to isolate and clone VEGF , reporting the isolation of a diffusible endothelial mitogen from conditioned medium by bovine pituitary follicular cells [2]. By the end of 1989, Ferrara reported the isolation of cDNA clones for bovine VEGF 164 and three human VEGF isoforms: VEGF 121, VEGF 165, and VEGF 189 [3]. Subsequently, Connolly et al. independently reported the isolation and sequencing of vascular permeability factor (VPF) [4]. cDNA cloning of VEGF [3] and VPF [5] revealed that VEGF and VPF are  the same molecule.

Ferrara’s laboratory has investigated many aspects of VEGF biochemistry/molecular biology, including the identification and characterization of its receptors (VEGFRs), regulation of VEGF activity by alternative RNA splicing and by extracellular proteolytic mechanisms, structure/function studies on the factor and its receptors, elucidation of its role in angiogenesis in bone and reproductive system.

In 1993, Ferrara’s laboratory reported that the murine anti-VEGF monoclonal antibody A4.6.1 exerted a potent inhibitory effect on the growth of three human tumor cell lines injected subcutaneously into nude mice [6]. These findings provided the first direct demonstration that inhibition of the action of an endogenous endothelial cell mitogen may result in suppression of tumor growth in vivo. Subsequently, Ferrara’s laboratory demonstrated that many other cell lines were inhibited in vivo by the anti-VEGF monoclonal antibody [7–11]. Intravital video microscopy techniques allowed a more direct verification of the hypothesis that anti-VEGF antibodies indeed block tumor angiogenesis [8]. Tumor spheroids were implanted in dorsal skinfold chambers inserted in nude mice. Noninvasive imaging of the vasculature revealed a complete suppression of tumor angiogenesis in anti-VEGF treated animals. Histological analysis showed a difference in the density of vascular elements between the two groups. Warren et al. [7] demonstrated that VEGF is a mediator of the in vivo growth of human colon carcinoma HM7 cells in a orthotopic nude mouse model of liver metastasis, and that treatment with anti-VEGF monoclonal antibody resulted in a decrease in the number, size, and vascularization of metastases. Three different human tumor cell lines were implanted in two locations in immunodeficient mice [12]. Treatment with the anti-VEGF monoclonal antibody was initiated when the tumor xenografts were already established and vascularized and resulted in time-dependent reductions in vascular permeability. These effects were accompanied by changes in the morphology of the vessels with reduction in diameter and tortuosity [12].

## The discovery of Bevacizumab

In 1997, the monoclonal antibody A4.6.1 was humanized to create Bevacizumab (Avastin, Genentech), an antibody suitable for clinical trials [13]. Bevacizumab binds and neutralizes all human VEGF-A isoforms and bioactive proteolytic fragments, but not mouse or rat VEGF, and inhibited the growth of human tumor cell lines in nude mice [13]. In 1997, Genentech initiated phase I clinical trials with Bevacizimab, showing that the antibody as a single agent was relatively nontoxic and that adding it to standard chemotherapy regimen did not significantly exacerbate chemotherapy-associated toxicities [14, 15]. In 1998, several phase II studies were initiated with Bevacizumab in different tumor types, either as single agent or in combination with chemotherapy. The most efficacy results were seen when Bevacizumab was combined with standard first-line chemotherapy in metastatic colorectal cancer [16], in stage IIIb/IV non-small cell lung cancer [17], and when was used as a single agent in renal cell cancer [18].

## FDA approval

These clinical trials t resulted in Food and Drug Administration (FDA) approval of Bevacizumab in February 2004 in a randomized double-blind phase III study in which it was administered in combination with bolus IFL (irinotecan, 5FU, leucovorin) chemotherapy as first-line therapy for previous untreated metastatic colorectal cancer [19]. Median survival was increased from 15.6 months in the bolus IFL + placebo arm to 20.3 months in the bolus IFL + Bevacizumab arm. Similar increases were seen in progression free survival (PFS), response rate, and duration of response. However, no other study conducted later reproduced the overall survival (OS) reported in the initial study. Treatment with  Bevacizumab is generally safe and well tolerated, but it can be accompanied by a variety of adverse effects, including hypertension (32% incidence), proteinuria (23% incidence), impaired wound healing (13% incidence), gastrointestinal perforation, bleeding, and thromboembolism, which are broadened or intensified by concurrent chemotherapeutic agents [20]. Hypertension occurs rapidly within hours or days after starting anti-VEGF therapy and is correlated with VEGF signaling inhibition. As compared to chemotherapy alone, the relative risk of bleeding on chemotherapy with Bevacizumab is 2–3 for most cancers.

In January 2005, Bevacizumab received approval from the European Medicine Agency (EMA) and in June 2006, the FDA approved its use in combination with5-FU-based chemotherapy as a second-line treatment for metastatic colorectal cancer [21]. In 2006, the FDA approved Bevacizumab for first-line treatment of advanced non-small cell lung cancer (NSCLC) in combination with standard chemotherapy carboplatin/paclitaxel [22]. In 2008, Bevacizumab was approved by the FDA for patients with metastatic breast cancer. Patients were randomized to receive paclitaxel alone or paclitaxel plus Bevacizumab. The addition of Bevacizumab to chemotherapy resulted in a 5.5-month survival in median PFS, but no statistically significant improvement in OS was observed [23]. Three further phase III trials of Bevacizumab in combination with chemotherapy in HER2-negative metastatic breast cancer demonstrated an extension of PFS, but no effects on OS, when compared to chemotherapy alone [24–26]. Because of these results, the FDA withdrew its approval for Bevacizumab in this indication in November 2011. Bevacizumab in combination with capecitabine is indicated for first-line treatment of adult patients with metastatic breast cancer in whom treatment with other chemotherapy options including taxanes or anthracyclines is not appropriate.

Bevacizumab was approved for use in first-line therapy of metastatic renal cell carcinoma with a 37% reduction in the risk of disease progression with the addition of Bevacizumab to interferon alpha (IFNα) compared with IFNα alone [27]. The efficacy of Bevacizumab in the first-line treatment in patients with cervical, ovarian,  fallopian tube, or peritoneal cancer was confirmed in other studies [28–30]. Bevacizumab was approved for the treatment of relapsed or progressive glioblastoma, demonstrating an improvement in PFS by 4.2 months when used alone and by 5.6 months when was combined with irinotecan [31].

Bevacizumab, in combination with carboplatin and paclitaxel is indicated for the front-line treatment of adult patients with advanced stages epithelial ovarian, fallopian tube, or primary peritoneal cancer. In combination with carboplatin and gemcitabine, it is indicated for the treatment of adult patients with first recurrence of platinum-sensitive epithelial ovarian, fallopian tube or primary peritoneal cancer who have not received prior therapy with bevacizumab or other VEGF inhibitors or VEGFRs targeted agents. Bevacizumab in combination with paclitaxel, topotecan, or pegylated liposomal doxorubicin is indicated for the treatment of adult patients with platinum-resistant recurrent epithelial ovarian, fallopian tube, or primary peritoneal cancer who received no more than two prior chemotherapy regimens and who have not received prior therapy with Bevacizumab or other VEGF inhibitors or VEGFRs targeted agents. In combination with paclitaxel and cisplatin or, alternatively, paclitaxel and topotecan in patients who cannot receive platinum therapy, is indicated for the treatment of adult patients with persistent, recurrent, or metastatic carcinoma of the cervix.

In 2020, the EMA approved the first biosimilar of Bevacizumab for the treatment of multiple types of cancer. The availability of biosimilars contributes to increased competition in the market and has the potential to enhance patient access to Bevacizumab therapy. The market for Bevacizumab biosimilars has grown significantly in the last few years. This increase can be ascribed to the high cost of the original medication as well as the rising incidence of cancer throughout the globe. The market is growing because of the launch of Bevacizumab biosimilars, which have given consumers and healthcare providers a more affordable option.

Furthermore, Bevacizumab has been recommended also by other organization guidelines, including The National Comprehensive Cancer Network (NCCN) and the European Society for Medical Oncology (ESMO), and reimbursement organizations, such as The National Institute for Health and Care Excellence (NICE) in the UK. NCCN recommends Bevacizumab in combination with paclitaxel for metastatic breast cancer, for NSCLC and glioblastoma [32–34]. EMSO recommends Bevacizumab in combination with other treatments in cervical, ovarian, and NSCLC . It is also may be used in combination with chemotherapy for metastatic colorectal cancer and in certain cases of hepatocellular carcinoma [35–39]. NICE recommends Bevacizumab for the treatment of advanced ovarian cancer [40].

The administration of Bevacizumab became popular among ophthalmologists for different diseases, such as choroidal neovascularization secondary to pathologic myopia or secondary to age-related macular degeneration, proliferative diabetic retinopathy, diabetic macular edema, retinopathy of prematurity, iris neovascularization, and neovascular glaucoma, and macular edema due to branch and central retinal vein occlusion.

The global Bevacizumab market was valued at approximately $8.1 billion in 2023 and is expected to reach around $12.5 billion by 2032. This market size growth is primarily driven by the increasing prevalence of chronic diseases such as cancer and ocular disorders, coupled with rising investment in healthcare infrastructure and advanced therapeutics.

## Criticisms and faithful

In most types of cancer, including breast, melanoma, pancreatic and prostate cancer, Bevacizumab failed to increase survival [41–44]. Moreover, bevacizumab did not extend survival in tumors considered “responsive” such as colon [45–47], breast [23], ovary [48–52], and renal cancers [53, 54]. Moreover, it has been demonstrated that the use of Bevacizumab had a detrimental effect on survival [55]. Despite impressive performances in animal models, however, inhibitors are not performing nearly as well in humans. Anti-angiogenic treatments lead to only 3–6 months increase in PFS, followed by a relapse in tumor angiogenesis and growth. When VEGF-targeted therapies are discontinued, the tumor vasculature is rapidly reestablished [56] and continuation of Bevacizumab treatment beyond progression was associated with greater benefit in terms of OS [57]. While Bevacizumab often improves PFS in cancer patients, this benefit doesn’t always translate into a corresponding increase in OS. This can be due to several factors, including the way tumors develop resistance to bevacizumab, the impact of subsequent therapies, and the statistical limitations of using PFS as a sole endpoint in clinical trials.

The limitations of applying Bevacizumab are attributed to drug resistance [58], metastasis promotion (while Bevacizumab is primarily used to inhibit tumor growth, some studies suggest it may have paradoxical effects in certain situations, potentially promoting metastasis under some circumstances [59]) and reduced delivery of chemotherapeutic agents, particularly in the early stages after administration, resulting from the dramatic decrease in tumor vasculature [60]. Preclinical studies have demonstrated that intrinsic resistance may result from an absence of VEGF or VEGFR in tumors from certain organ sites [61]. A tumor may exhibit acquired resistance by co-opting existing blood vessels from vasculature rich organs, such as the lungs, liver or brain [62].

The inhibition of VEGF and VEGFRs act by selectively blocking the formation of immature blood vessels but leaving behind the mature and functional vasculature (normalization effect) [63, 64].

The development of hypoxia-resistant tumor subpopulations which can outgrow the sensitive tumor cells [65] or the selection of more mature, stable vessels that are intrinsically less responsive to anti-angiogenic treatment [66] could also cause resistance. The hypoxic environment results in the upregulation of other pro-angiogenic factors, including fibroblast growth factor 2 (FGF-2), platelet derived growth factor (PDGF), hepatocyte growth factor (HGF), interleukin 8 (IL-8). which do not respond to Bevacizumab [67]. Bevacizumab can induce tumor hypoxia, potentially affect its effectiveness and lead to paradoxical effects. While Bevacizumab can inhibit tumor growth by targeting VEGF, it can also disrupt tumor vasculature, leading to decreased oxygen levels within the tumor (hypoxia). This hypoxia can, in turn, promote cancer progression and resistance to treatment [68].

In this context, a variety of multi-targeted angiogenesis inhibitors have been developed, including Sorafenib, Sunitinib, Pazopanib, Vandetanib, Axitinib, Regorafenib, Cabizantinib, and Lenvatinib approved by the FDA for application in patients with various cancers.

Tumor-associated endothelial cells may also be a source of resistance due to various cytogenetic abnormalities [69].

Numerous clinical trials have been developed including combination of Bevacizumab with chemotherapy, radiotherapy, immunotherapy, and cancer vaccines [70]. A significant and fascinating story is the combination of Bevacizumab with immunotherapy. Several clinical trials evaluating the combination of programmed death ligand-1 (PD-L1) (e.g., atezolizumab, durvalumab, avelumab), programmed death-1 (PD-1) (e.g., nivolumab, pembrolizumab), or cytotoxic T-lymphocyte antigen-4 (CTLA-4) (e.g., ipilimumab, tremelimumab) inhibitors with  Bevacizumab are ongoing across various tumor types [71]. A clinical study of combination therapy using the anti-CTLA-4 antibody ipilimumab with Bevacizumab reported efficacy in patients with advanced melanoma resulting in a median OS of more than 2 years [72]. In 2020, the FDA approved the PD-L1 monoclonal antibody atezolimumab combined with Bevacizumab as first line therapy for unresectable or metastatic hepatocellular carcinoma [73]. Clinical trials for glioblastoma multiforme (GBM) are exploring the combination of Bevacizumab with immune checkpoint inhibitors. While Bevacizumab has shown some benefit in improving PFS and delaying quality of life decline, it has not significantly extended OS in phase III trials. Immunotherapy, including checkpoint inhibitors like nivolumab has shown promise in some trials, but results have been mixed, with some studies showing no significant improvement in survival [74]. A retrospective case series used single agent nivolumab to treat progression after bevacizumab in patients with glioblastoma [75]. This retrospective study found a minimal benefit in patients with recurrent glioblastoma after progression on  Bevacizumab. Due to the lack of clear evidence regarding the best treatment for such patients, post- Bevacizumab therapy also represents an unmet need in neuro-oncology for patients with refractory gliobastoma. Bispecic antibodies are man-made antibody-based molecules with two different antigen-binding sites that were first described and identified more than 50 years ago. The stable bispecific antibody HB0025, which targets both PD-L1 and VEGF, while Bevacizumab specifically targets VEGF. Preclinical studies suggest HB0025 may be more effective than   Bevacizumab and other treatments in inhibiting tumor growth [76]. Finally, Bevacizumab is also an example of application of precision medicine. While Bevacizumab is widely used, research is ongoing to identify predictive factors for treatment response, including biomarkers, pharmacogenomics, and therapeutic drug monitoring, to optimize its use in precision medicine. Bevacizumab plays a role in precision medicine by targeting a key pathway involved in tumor growth and metastasis. While not a direct biomarker-driven therapy, bevacizumab’s effectiveness can be influenced by various factors, including tumor type, stage, and patient characteristics, making its use somewhat tailored to individual patient profiles. For example, in metastatic colorectal cancer, Bevacizumab’s effectiveness can be influenced by RAS and BRAF mutations. In left-sided RAS wild-type tumors, bevacizumab with chemotherapy can be a treatment option [77]. In advanced liver cancer, Bevacizumab is used with atezolizumab, and the combination’s effectiveness can be influenced by factors like albumin-bilirubin grade and the presence of metastases [78].

Increased understanding of tumor genetics and molecular markers has expanded the treatment options for patients as established by the NCCN guidelines recommend that patients receive treatment with Bevacizumab [79].

## Conflict of interest

The authors declare no competing interests.

## Data Availability

No datasets were generated or analysed during the current study.
